# Machine learning-based radiomics for differentiating lung cancer subtypes in brain metastases using CE-T1WI

**DOI:** 10.3389/fonc.2025.1599882

**Published:** 2025-06-19

**Authors:** Xueming Xia, Wei Du, Qiheng Gou

**Affiliations:** ^1^ Division of Head & Neck Tumor Multimodality Treatment, Cancer Center, West China Hospital, Sichuan University, Chengdu, China; ^2^ Department of Targeting Therapy & Immunology, Cancer Center, West China Hospital, Sichuan University, Chengdu, China

**Keywords:** radiomics, magnetic resonance imaging, lung cancer, brain metastases, machine learning

## Abstract

**Objectives:**

The purpose of this research was to create and validate radiomic models based on machine learning that can effectively discriminate between primary non-small cell lung cancer (NSCLC) and small cell lung cancer (SCLC) in individuals with brain metastases (BMs) by utilizing high-dimensional radiomic characteristics derived from contrast-enhanced T1-weighted imaging (CE-T1WI).

**Methods:**

A cohort of 260 individuals were chosen as participants. Among them, 173 individuals had NSCLC with 228 BMs, while 87 patients were diagnosed with SCLC with 142 BMs. Patients were allocated to a training dataset with a total of 259 BMs and an independent test dataset with a total of 111 BMs. Tumor tissues in axial CE-T1WI were manually outlined to delineate regions of interest (ROIs). Radiomic features were obtained from the ROIs using PyRadiomics, which were then chosen through a multistep selection process, including least absolute shrinkage and selection operator (LASSO) regression. Ten machine learning models, including Light Gradient Boosting Machine (LightGBM), RandomForest, and eXtreme Gradient Boosting (XGBoost), were built using selected features. The models’ performance was evaluated using receiver operating characteristic (ROC) analysis and area under the curve (AUC) calculations, complemented by additional metrics such as accuracy, specificity, sensitivity, positive predictive value (PPV), and negative predictive value (NPV).

**Results:**

A total of 833 radiomic features were extracted from the ROIs. Through a multistep selection process, a refined subset of 15 optimal radiomic features was identified for model training. Ten classifier models were built based on features extracted from CE-T1WI. In the training dataset, the top-performing classifiers were the XGBoost, LightGBM, support vector machine (SVM) and random forest models, which achieved AUC of 0.963, 0.881, 0.876 and 0.855, respectively, with 5-fold cross-validation. Among the ten models tested, the LightGBM algorithm exhibited superior performance, with an AUC of 0.853 in the test cohort. This performance was superior to that of other models, such as RandomForest (AUC 0.843) and ExtraTrees (AUC 0.835). Radiomic features significantly contributed to the differentiation between NSCLC and SCLC.

**Conclusion:**

Machine learning-based radiomics using CE-T1WI data is highly effective in distinguishing between NSCLC and SCLC in patients with BMs. The LightGBM model showed the best performance, suggesting that this approach shows promise as a supportive, non-invasive diagnostic tool, pending further validation in prospective clinical settings.

## Introduction

Brain metastases (BMs) are the most common form of malignant neoplasms affecting the brain, representing a significant complication in cancer progression and management (1). Nearly 40% of cancer patients are estimated to develop BMs during the course of their illness, which markedly increases mortality rates and severely compromises quality of life (2). The growing incidence of BMs in recent years is largely attributable to advances in systemic anticancer therapies that prolong patient survival, the unique immunological microenvironment of the brain, and enhanced detection capabilities through magnetic resonance imaging (MRI) (3). Among all primary tumors, advanced lung cancer (LC)—comprising non-small cell lung cancer (NSCLC) and small cell lung cancer (SCLC)—accounts for approximately 50% of all BM cases (4). Notably, about 25% to 50% of NSCLC patients and nearly half of SCLC patients develop BMs during the disease course (5). In particular, NSCLC patients with specific genetic mutations or elevated programmed cell death ligand 1 (PD-L1) expression benefit significantly from targeted therapy and immunotherapy, which have enhanced overall survival in recent years (6, 7). Consequently, early and accurate differentiation between NSCLC and SCLC patients with BMs is essential for tailoring appropriate treatment strategies and improving clinical outcomes.

Biopsy, while an effective traditional diagnostic method, often requires invasive procedures and carries the risk of severe complications, especially when involving brain lesions. Moreover, brain lesion biopsies have limited ability to accurately differentiate between the two subtypes of LC (8). This challenge is particularly evident in patients who present with both a lung mass and brain metastases at initial diagnosis and are in poor general condition, making them unsuitable candidates for biopsy. In such cases, a non-invasive diagnostic approach is critically important. MRI, with its exceptional soft-tissue contrast and detailed anatomical information, is a cornerstone for evaluating BMs, and contrast-enhanced T1-weighted imaging (CE-T1WI) is crucial for detecting them (9). BMs from NSCLC and SCLC exhibit distinct, though occasionally overlapping, MRI features (10). NSCLC-related BMs typically present as solitary or fewer, larger lesions with well-defined margins, marked contrast enhancement, and significant peritumoral edema (11). Certain NSCLC subtypes, such as adenocarcinoma, may exhibit distinctive imaging features, though considerable variability exists. In contrast, SCLC-related BMs usually appear as multiple small, densely clustered lesions with intense enhancement and surrounding edema, often with poorly defined boundaries due to rapid tumor growth (12). However, overlapping features—particularly in enhancement patterns and edema—can make it challenging to reliably distinguish between NSCLC and SCLC metastases based solely on MRI.

Recently, advancements in radiomics and machine learning (ML) have shown promise in overcoming this limitation. Radiomics, which involves deriving quantitative features from imaging data, has gained significant attention for its ability to uncover latent patterns and provide valuable diagnostic and prognostic insights (13). ML algorithms can analyze these complex features, enabling the differentiation between SCLC and NSCLC by identifying subtle image characteristics that may not be detectable through conventional imaging analysis alone. Currently, various studies have aimed to develop non-invasive ML or deep learning (DL) techniques that utilize features extracted from MRI to assess the characteristics of BMs and determine their origins. Using a DL model, Sui L et al. reported an AUC ranging from 0.8024 to 0.8019 for subtype classification of lung cancer patients with BMs (14). Egashira M et al. found that the AUC of the ML model was 0.744 for NSCLC and 0.861 for SCLC (15). However, previous studies have encountered several limitations. First, these radiomic models generally exhibited low AUCs and demonstrated relatively poor diagnostic performance. Second, although radiomics-based classification models have shown efficacy in tumor type estimation, there has been limited focus on predicting the origins of BMs.

This study addresses a critical clinical need by investigating a non-invasive machine learning-based approach to accurately differentiate NSCLC from SCLC in patients with BMs. By enhancing diagnostic precision without the need for traditional biopsy, this method holds promise as a supportive tool in facilitating personalized treatment planning and improving patient outcomes.

## Materials and methods

### Patient and MRI protocol

This research involved a group of patients confirmed with BMs originating from the LC at our institution between January 2021 and December 2023. The research was carried out following the guidelines of the Declaration of Helsinki. The institutional research ethics committee reviewed the study and waived the requirement for formal approval, as it involved retrospective analysis of anonymized patient data. The requirements for participating in the study included the following: 1) histopathologically confirmed diagnosis of LC; 2) diagnosis of BMs by pathology or MRI; and 3) availability of pretreatment contrast-enhanced MRI scans of the head. Three factors were excluded: 1) pre-MRI treatment with antitumor therapy, such as radiotherapy, targeted therapy or immunotherapy; 2) BMs less than one centimeter in diameter; and 3) substantial artifacts in the images. A total of 260 patients were retrospectively identified, with 87 patients with SCLC and 173 patients with NSCLC.

All participants were examined using Siemens Trio scanners (3.0T) at the institution’s radiology department. Gadopentetate dimeglumine (0.1 mmol/kg), which was calculated based on weight, was injected intravenously into patients. The imaging parameters for CE-T1WI acquisition were as follows: TR/TE/TI = 1900/2.26/900 ms, flip angle = 9°, slice thickness = 1 mm, axial field of view (FOV) = 25.6 × 25.6 cm², and data matrix = 256 × 256. CE-T1WI were acquired in multidirectional mode within a 90–250 second interval.

### Data preprocessing and image segmentation

The dataset was split 7:3 between training and test sets for model evaluation. BMs from the same patient were not split between the training and validation cohorts. Each patient’s lesions were exclusively assigned to either the training or the validation set to ensure independence between cohorts and to avoid any potential data leakage that could artificially inflate model performance. Medical imaging datasets often present heterogeneous voxel spacing due to differences in scanner models and acquisition protocols. To address this variability, a fixed-resolution resampling approach was employed in this study, standardizing all images to an isotropic voxel size of 1×1×1 mm. This ensured uniform spatial resolution across the dataset. Subsequently, z-score normalization (zero-mean, unit-variance) was applied to harmonize feature scaling. In addition, bias field correction using the N4ITK algorithm was performed to mitigate intensity inhomogeneities resulting from magnetic field non-uniformities.

In this research, the 3D Slicer open-source software application (Version 4.11), accessible at https://download.slicer.org, was utilized to assist in the three-dimensional manual segmentation of MRI. Two seasoned radiologists, who were unaware of the histopathological information, independently performed the image segmentation. Radiologist A, who has seven years of expertise in brain imaging, manually outlined the tumor tissues in the axial CE-T1WI. The radiologist meticulously outlined each image layer to precisely identify and segment the tumors following the software’s designated protocol. The delineation of regions of interests (ROIs) in each slice was based on the boundaries of the tumor tissue, encompassing necrotic areas and tumor blood vessels while excluding peritumoral edema. Radiologist B, who has a decade of expertise in neuroimaging, thoroughly examined all the ROIs delineated by Radiologist A to ensure precision and uniformity. Conflicts were settled through discussion until a consensus was reached. Since the segmentation was performed using a single set of delineations validated by expert consensus, inter- and intra-observer variability metrics—such as the Dice similarity coefficient and intraclass correlation coefficient (ICC)—were not evaluated. This approach aligns with common practices in radiomics studies when multi-reader segmentations are unavailable, aiming to reduce variability through stringent expert validation.

### Radiomic feature extraction and selection

A total of 833 radiomic features were obtained from the ROIs on the CE-T1WI using PyRadiomics (Version 3.0.1) in Python 3.9 (16). These features can be classified into three groups: geometry, intensity, and texture, which describe the shape, statistical distribution, and spatial patterns of the tumor, respectively.

The Mann–Whitney U test and feature screening were carried out, and only characteristics with a p value below the 0.05 threshold were retained. Spearman’s rank correlation coefficient was used to analyze the relationships between characteristics with considerable reproducibility. Characteristics with a correlation coefficient exceeding 0.9 between any two features were selected by using a greedy recursive deletion method. The least absolute shrinkage and selection operator (LASSO) regression model was employed to build a signature by shrinking regression coefficients toward zero and setting many unrelated features to zero with reference to the regulation weight λ (17). The optimal value of λ was found through cross-validation with ten folds, resulting in a radiomic signature with retained features having nonzero coefficients.

### Radiomic model building

The selected features were utilized as input variables for model development (18). A total of ten machine learning algorithms were evaluated to identify the most effective classifier. The dataset was partitioned into training and testing sets in a 7:3 ratio. Cross-validation was employed during the training phase to optimize hyperparameters and evaluate model stability. The entire training set was used to build the predictive models, while the test set served as an independent dataset to assess model performance. Hyperparameter tuning was conducted via grid search to determine the optimal configuration for each algorithm. The final classifier was selected based on its performance metrics, including accuracy, sensitivity, specificity, and area under the receiver operating characteristic curve (AUC), as evaluated on the validation set. To further assess clinical utility, decision curve analysis (DCA) was performed, and the standardized net benefit (sNB)—ranging from 0 to 1—was calculated.

### Statistical analysis

Python (Version 3.9) was used for the statistical analysis (19). The threshold for significance was established at a p value of less than 0.05. Group differences were measured by employing t tests or Mann–Whitney U tests for quantitative metrics and chi–square or Fisher’s exact tests for qualitative metrics. To compare the performance of different machine learning models, DeLong’s test was applied to assess the statistical significance of differences between correlated receiver operating characteristic (ROC) curves.

## Results

### Patient characteristics

**Figure 1:**
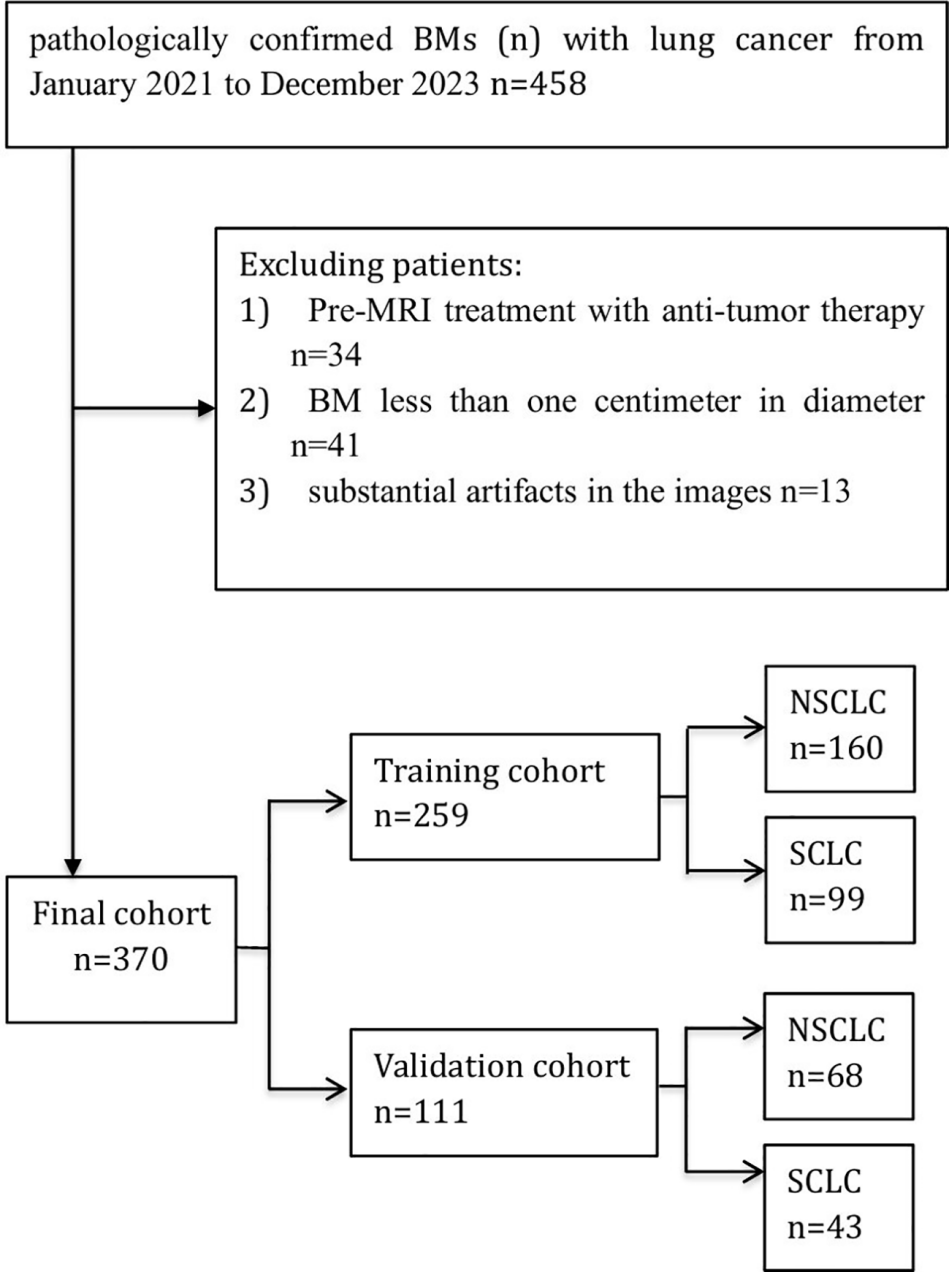
Flow diagram of the study enrollment BMs (n) with lung cancer. Of 458 patients with pathologically confirmed brain metastases from lung cancer (Jan 2021–Dec 2023), 88 were excluded. The final cohort (n = 370) was split into a training set (n = 259) and a validation set (n = 111), with subgroups of NSCLC and SCLC.

**Figure 1** illustrates the flowchart for the choice of BMs for LC patients. A cohort of 260 individuals who received at our institution from January 2021 to December 2023 were chosen as participants for this investigation in accordance with the predefined inclusion and exclusion criteria. Among them, 173 individuals had NSCLC with 228 BMs, while 87 patients were diagnosed with SCLC with 142 BMs. Patients were allocated to a training dataset with a total of 259 BMs and an independent test dataset with a total of 111 BMs. A pathologist reviewed the pathological data. **Table 1** provides a comprehensive overview of the patient traits. Notably, no meaningful clinical differences were detected between the training and validation groups.

**Table 1:** Characteristics of the included patients with BMs (n).

Characteristics	Training cohort (n = 259)			Validation cohort (n =111)			P*
	NSCLC (n=160)	SCLC(n=99)	P	NSCLC (n=68)	SCLC (n=43)	P	
Gender (%)			0.242			0.842	0.791
Male	95 (59.4%)	60 (60.6%)		38 (55.9%)	22 (51.2%)		
Female	65 (40.6%)	39 (39.4%)		30 (44.1%)	21 (48.8%)		
Age, mean ± SD (years)	61.5 ± 8.8	63.5 ± 8.0	0.70	62.3 ± 7.1	62.5 ± 7.3	0.213	0.802
Median age (years)	61.0 (47-81)	62.4 (46-77)		62.1 (46-79)	62.9 (45-80)		

P is obtained from the chi-squared test or Fisher’s exact test comparing patients with NSCLC and SCLC in both the training and validation cohorts, respectively. P* denotes the discrepancy of every factor between the training and validation cohorts. NSCLC, non-small cell lung cancer; SCLC, small cell lung cancer; SD, standard deviation.

### Feature selection and model construction

**Figure 2:**
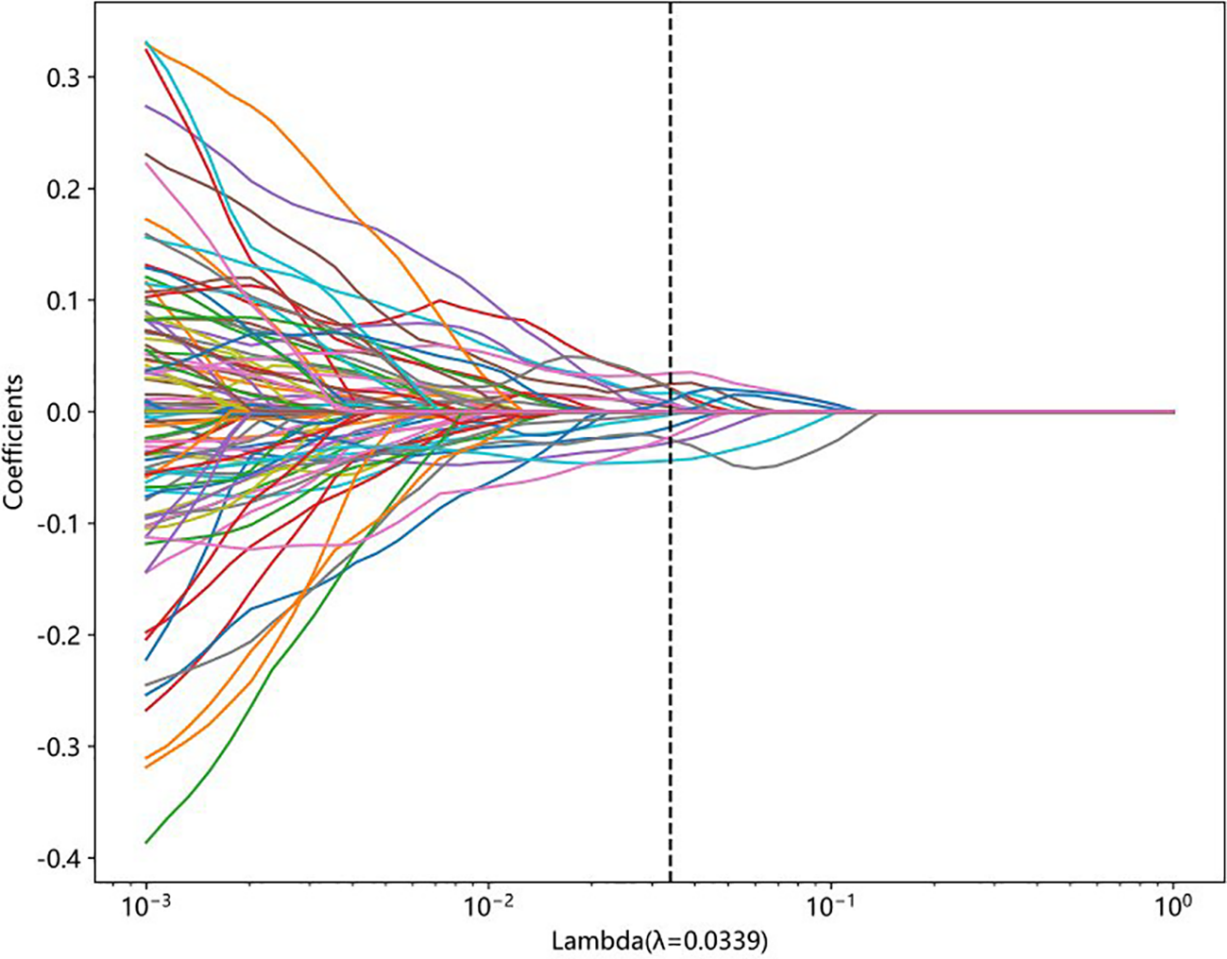
LASSO coefficient profiles of features generated from 10-fold cross-validation. Each colored line represents the trajectory of a feature’s coefficient as the regularization parameter lambda changes. The vertical dashed line indicates the optimal lambda value (λ = 0.0339), at which the model achieves the best balance between sparsity and performance.

**Figure 3:**
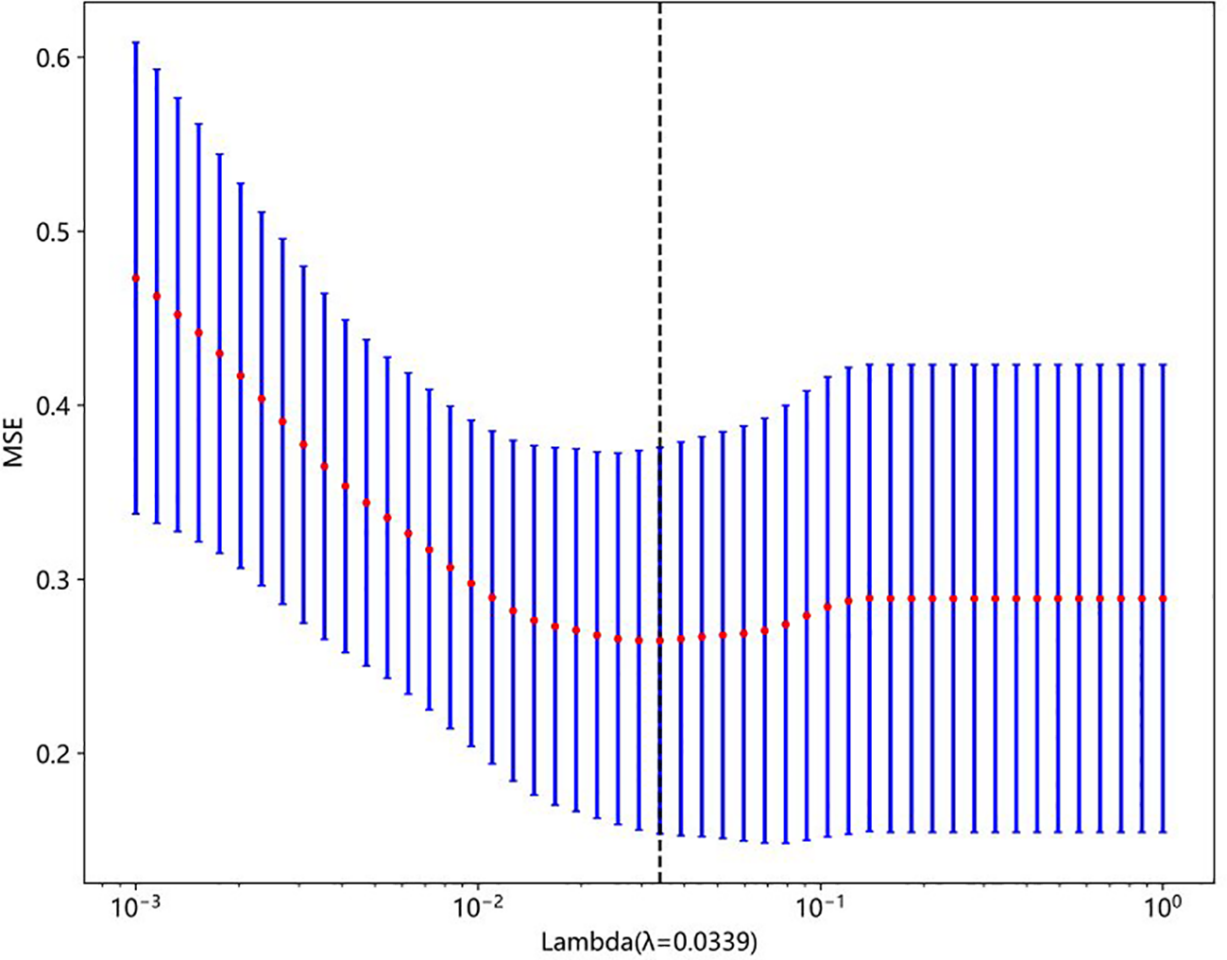
Mean squared error (MSE) plot from 10-fold cross-validation used to select the optimal regularization parameter (lambda) in LASSO regression. The red dots represent the average MSE for each lambda, and the blue bars show ±1 standard error. The vertical dashed line indicates the optimal lambda value (λ = 0.0339), which minimizes the prediction error.

**Figure 4:**
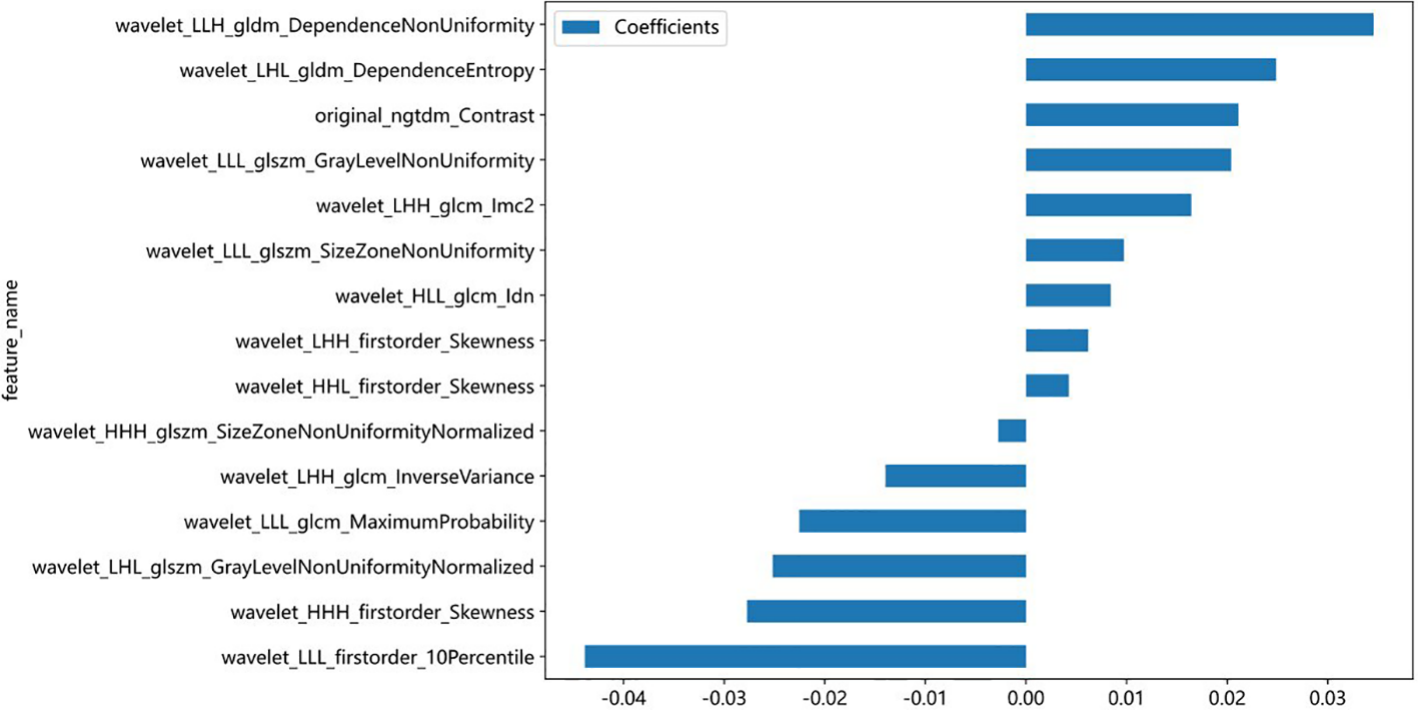
Bar plot showing the coefficients of the 15 most predictive radiomic features selected by the LASSO model. Positive coefficients indicate a direct association with the outcome, while negative coefficients suggest an inverse relationship. Feature names reflect the original or wavelet-transformed image filters and statistical classes used in radiomic extraction.

A total of 833 handcrafted features were extracted, with 538 features chosen through statistical tests. A total of 117 features were then selected based on correlation and a recursive deletion strategy. Fifteen optimal radiomic features were selected using the LASSO logistic model and regularization parameter λ with coefficients and mean standard errors shown in **Figures 2**, **3**. A Rad signature was established by utilizing the non-zero coefficients features selected by LASSO regression and features coefficients are displayed in **Figure 4**. The multistep selection process resulted in a final subset of 15 radiomic features, which were subsequently used for model training, as shown in **Table 2**. These selected features demonstrated a strong discriminatory ability between the two lung cancer subtypes in the training group as well as the testing group. Ten machine learning algorithms were evaluated to determine the most effective classifier.

**Table 2:** Radiomics features obtained by LASSO regression analysis.

Sequence	Radiomics features
1	original_ngtdm_Contrast
2	wavelet_HHH_firstorder_Skewness
3	wavelet_HHH_glszm_SizeZoneNonUniformityNormalized
4	wavelet_HHL_firstorder_Skewness
5	wavelet_HLL_glcm_Idn
6	wavelet_LHH_firstorder_Skewness
7	wavelet_LHH_glcm_Imc2
8	wavelet_LHH_glcm_InverseVariance
9	wavelet_LHL_gldm_DependenceEntropy
10	wavelet_LHL_glszm_GrayLevelNonUniformityNormalized
11	wavelet_LLH_gldm_DependenceNonUniformity
12	wavelet_LLL_firstorder_10Percentile
13	wavelet_LLL_glcm_MaximumProbability
14	wavelet_LLL_glszm_GrayLevelNonUniformity
15	wavelet_LLL_glszm_SizeZoneNonUniformity

### Performance of the models

**Figure 5:**
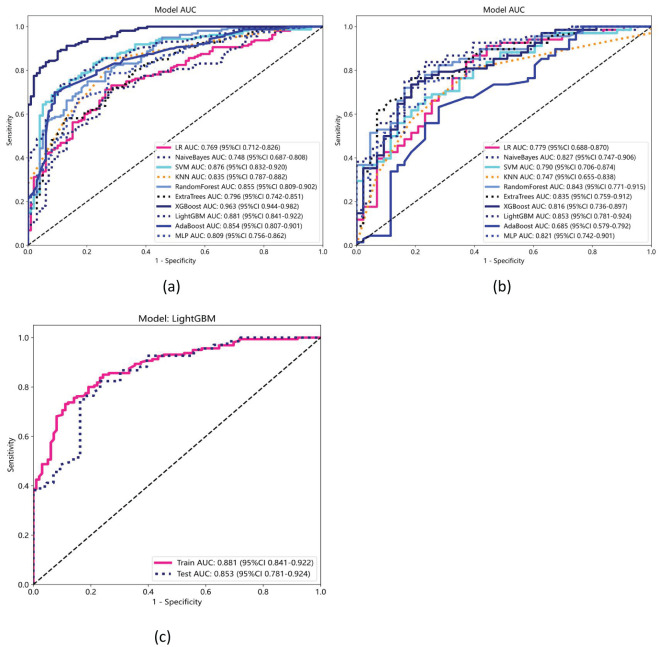
Receiver operating characteristic (ROC) curves for ten machine learning models in the **(a)** training and **(b)** testing datasets. **(c)** ROC curves for the LightGBM model, which achieved the highest performance, with an AUC of 0.881 (95% CI: 0.841–0.922) in the training set and 0.853 (95% CI: 0.781–0.924) in the testing set.

**Figure 6:**
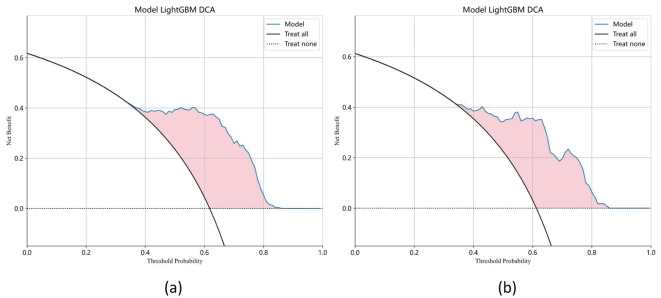
Decision curve analysis (DCA) of the LightGBM model in the training **(a)** and test cohorts **(b)**. The x-axis represents the threshold probability, and the y-axis indicates the net benefit. The LightGBM model demonstrates a higher standardized net benefit (sNB) across a range of clinically relevant threshold probabilities, indicating superior clinical utility.

Ten classifier models were built based on features extracted from MRI. The classifiers exhibited robust efficiency in relation to the AUC and accuracy. In the training dataset, the top-performing classifiers were the XGBoost, LightGBM, SVM and RandomForest models, which achieved AUCs of 0.963, 0.881, 0.876 and 0.855, respectively, with 5-fold cross-validation. In addition, these classifiers also demonstrated favorable performance in the independent test. In the testing dataset, LightGBM achieved the highest AUC of 0.853, significantly outperforming random forest (0.843), extra trees (0.835), and naive Bayes (0.827), with differences confirmed as statistically significant by DeLong’s test (p < 0.05). The ROC contours for these models were displayed in **Figure 5**, and a thorough contrast was presented in **Table 3**. DCA of LightGBM in the training and test cohorts is shown in **Figure 6**.

**Table 3:** Performance of ten machine learning models in the training and validation sets.

Model name	Accuracy	AUC	95% CI	Sensitivity	Specificity	PPV	NPV	Task
LR	0.718	0.769	0.7116- 0.8262	0.706	0.737	0.813	0.608	label-train
LR	0.757	0.779	0.6879 - 0.8695	0.853	0.605	0.773	0.722	label-test
NaiveBayes	0.714	0.748	0.6869 - 0.8083	0.712	0.717	0.803	0.607	label-train
NaiveBayes	0.802	0.827	0.7472 - 0.9060	0.824	0.767	0.848	0.733	label-test
SVM	0.792	0.876	0.8316 - 0.9200	0.750	0.859	0.896	0.680	label-train
SVM	0.703	0.790	0.7063 - 0.8737	0.662	0.767	0.818	0.589	label-test
KNN	0.695	0.835	0.7868 - 0.8822	0.587	0.869	0.879	0.566	label-train
KNN	0.667	0.747	0.6550 - 0.8382	0.574	0.814	0.830	0.547	label-test
RandomForest	0.764	0.855	0.8090 - 0.9016	0.744	0.798	0.856	0.658	label-train
RandomForest	0.757	0.843	0.7707 - 0.9150	0.706	0.837	0.873	0.643	label-test
ExtraTrees	0.687	0.796	0.7417 - 0.8511	0.569	0.879	0.883	0.558	label-train
ExtraTrees	0.775	0.835	0.7589 - 0.9121	0.750	0.814	0.864	0.673	label-test
XGBoost	0.888	0.963	0.9443 - 0.9816	0.881	0.899	0.934	0.824	label-train
XGBoost	0.757	0.816	0.7361 - 0.8966	0.721	0.814	0.860	0.648	label-test
LightGBM	0.788	0.881	0.8410 - 0.9216	0.725	0.889	0.913	0.667	label-train
LightGBM	0.793	0.853	0.7809 - 0.9243	0.809	0.767	0.846	0.717	label-test
AdaBoost	0.776	0.854	0.8075 - 0.9007	0.694	0.909	0.925	0.647	label-train
AdaBoost	0.640	0.685	0.5790 - 0.7917	0.588	0.721	0.769	0.525	label-test
MLP	0.757	0.809	0.7560 - 0.8622	0.775	0.727	0.821	0.667	label-train
MLP	0.748	0.821	0.7416 - 0.9007	0.735	0.767	0.833	0.647	label-test

LR, logistic regression; SVM, support vector machine; KNN, k-nearest neighbors; XgBoost, eXtrema gradient boosting; LGBM, light gradient boosting machine; AdaBoost, adaptive boosting; MLP, multilayer perceptron; PPV, positive predictive value; NPV, negative predictive value.

## Discussion

This research intended to evaluate the viability and precision of employing ML techniques to differentiate between NSCLC patients and SCLC patients with BMs by utilizing high-dimensional radiomic features derived fromCE-T1WI. Ten distinct machine learning prediction models were constructed and evaluated. Notably, the LightGBM algorithm exhibited superior performance, attaining an AUC of 0.853 in the test cohort. Consequently, the findings suggest promising prospects for ML models in predicting tumor pathology types.

Lung cancer subtype classification is critical for determining treatment strategies, as NSCLC and SCLC have different treatment protocols and prognoses (20). SCLC is an aggressive disease with a lack of predictive biomarkers, and treatment primarily involves chemotherapy and radiotherapy (21). NSCLC can be treated with surgery combined with radiotherapy and chemotherapy. Additionally, targeted therapies based on driver genes and immunotherapy with immune checkpoint inhibitors have significantly improved NSCLC patient survival (22). Histopathological examination is the best way to diagnose brain tumors, but brain biopsies are associated with risks such as bleeding and damage to critical brain tissue (23). This is a concern for patients who are not suitable for biopsy or those with severe comorbidities. CE-T1WI is crucial for detecting BMs, but its ability to differentiate the origin of BMs is limited. Non-invasive diagnostic methods using radiomics and ML show promise for improving clinical decision-making and identifying candidates for targeted therapy and immunotherapy. Pathological transformation between SCLC and NSCLC during treatment may impact treatment strategies (24). A study by Thai et al. reviewed the evolving landscape of lung cancer subtypes and the corresponding imaging and treatment strategies, reinforcing the necessity of precise subtype differentiation for optimized therapeutic outcomes (20). Finally, Vogelbaum et al. provided guidelines for treating BMs, reinforcing the importance of precise diagnostic tools to guide treatment strategies (25). Some studies have demonstrated that the ML technique can offer additional dynamic diagnostic capabilities and valuable diagnostic insights that traditional imaging might miss (26, 27).

Previous research has extensively investigated the differentiation of BMs originating from NSCLC and SCLC utilizing a variety of methodologies, with a primary focus on DL and ML techniques. Sui et al. employed DL models on CE-T1WI, achieving AUCs of 0.8024 for NSCLC and 0.8019 for SCLC, highlighting the possibility of DL within this field (14). Similarly, Egashira et al. showed an AUC of 0.744 for NSCLC and 0.861 for SCLC using ML models, emphasizing the significance of radiomics features in predictive modeling (15). Notably, a related study indicated that a model using individual radiomic features derived from CE-T1WI with the Xgboost algorithm achieved optimal performance, with an AUC of 0.85 in inner testing and 0.80 in exterior testing (28). In another study, the proposed radiomics-based classifier achieved a sensitivity of 94.44% and specificity of 95.33%, outperforming DL-based classifiers and demonstrating that the radiomic approach is a valuable diagnostic tool for differentiating lung cancer subtypes in BMs with small datasets (29).

Our findings align with the aforementioned research by demonstrating the efficacy of machine learning-based radiomics on CE-T1WI in differentiating between NSCLC and SCLC in patients with BMs. CE-T1WI is capable of providing detailed insights into the pathological structure of cancers (30, 31). The LightGBM algorithm in our study achieved an AUC of 0.853 in the validation cohort, which is similar to or exceeds that of models reported in previous research (28, 32). This consistency underscores the robustness of ML approaches in handling high-dimensional radiomic features for accurate tumor classification. However, our study diverges from previous research in several key aspects. Unlike Sui et al. and Egashira et al., who reported lower AUCs, our study achieved higher predictive accuracy, potentially due to the incorporation of a larger and more diverse dataset, as well as the use of advanced feature selection techniques such as LASSO regression. Moreover, our focus on comprehensive ML model evaluation, including DCA, provided a further rigorous examination of model capability, highlighting the clinical utility of our approach.

In the present study, ten distinct ML models were developed and evaluated, and their performances were assessed using various metrics. Of these models, LightGBM exhibited the best performance, achieving an AUC of 0.853 in the validation cohort. This was followed by the random forest, extra trees, and naive Bayes models, which achieved AUCs of 0.843, 0.835, and 0.827, respectively. **Table 3** and **Figure 5** show the performances of the models, highlighting the superior diagnostic capabilities of the LightGBM model on both the training and test datasets. The efficiency of the LightGBM model in dealing with extensive data and multidimensional features contributes to its strong performance. The algorithm effectively prevents overfitting with techniques such as gradient-based one-sided sampling (GOSS) and exclusive feature bundling (EFB), ensuring high accuracy and robustness in predictions. Advanced feature selection methods such as LASSO regression focus on relevant features, improving the model’s ability to differentiate between SCLC and NSCLC. The observed discrepancy between the XGBoost model’s AUC of 0.963 in the training dataset and 0.816 in the validation dataset suggests potential overfitting. This significant drop in performance indicates that while XGBoost effectively captures patterns in the training data, its generalizability to unseen data is limited. Overfitting may arise due to the model’s complexity or insufficient regularization, causing it to memorize training data rather than learn generalizable features. This highlights that advanced techniques like GOSS and EFB used by LightGBM help mitigate overfitting, suggesting that similar strategies might improve XGBoost’s performance. Additionally, the absence of precision-recall curves in the study could obscure the model’s performance on imbalanced datasets, which may contribute to the observed AUC drop in the validation set. To address this, future work could incorporate regularization techniques, automated feature selection like LASSO regression, or cross-validation adjustments to enhance XGBoost’s robustness and generalizability.

We extracted 833 radiomic features from axial CE-T1WI using the PyRadiomics package (33) and identified 15 key features that effectively differentiated SCLC from NSCLC. These included intensity, texture, and geometric descriptors such as original_ngtdm_Contrast, wavelet_HHH_firstorder_Skewness, and wavelet_LLL_glcm_MaximumProbability. These features have biological relevance (34, 35). For example, original_ngtdm_Contrast reflects tumor heterogeneity, which is typically higher in SCLC due to its rapid proliferation, necrosis, and disorganized cellular architecture. The wavelet_HHH_firstorder_Skewness captures asymmetrical intensity distributions, potentially linked to irregular tumor growth in SCLC. In contrast, NSCLC often exhibits more structured, homogeneous patterns. Geometric features provide insight into tumor shape and invasive potential. SCLC frequently presents with irregular, infiltrative borders, while NSCLC tends to show more defined and organized structures. These differences in morphology and biological behavior are well represented by the selected radiomic features and enhance the model’s ability to distinguish between subtypes (36). These features are crucial for accurate classification and, when combined with advanced ML algorithms, improve the ability to differentiate between LC subtypes for better diagnosis and treatment planning (37).

This study has several limitations that should be acknowledged. First, the retrospective design and limited sample size may affect the generalizability of our findings. Future multicenter prospective studies incorporating deep learning techniques could enhance the model’s robustness and clinical applicability. Second, our analysis relied solely on CE-T1WI sequences; incorporating additional sequences (e.g., T2-FLAIR) might improve discriminatory performance. Third, although SHAP (SHapley Additive exPlanations) analysis was not performed in this study, we acknowledge its value in improving model transparency. SHAP could help visualize the impact of each feature on model predictions, offering deeper insights into the decision-making process (38, 39). We consider this an important direction for future work to enhance interpretability and clinical trust. Fourth, class imbalance in the training dataset and the absence of precision-recall curves may obscure the model’s true performance on minority classes. These curves are essential for evaluating imbalanced datasets and should be included in future work. Fifth, manual tumor segmentation may introduce inter-observer variability; automated or semi-automated methods could improve reproducibility. Finally, while our top-performing models demonstrated strong AUCs, clinical translation would further require validating the model across diverse, real-world datasets to ensure practical utility.

## Conclusion

Our research illustrates that the application of machine learning-based radiomics, utilizing high-dimensional features extracted from CE-T1WI, is a highly effective method for distinguishing between primary NSCLC and SCLC in patients with BMs. The LightGBM model displayed superior capability with an AUC of 0.853 in the test cohort, suggesting considerable promise for enhancing diagnostic precision and facilitating the refinement of customized protocol. This machine learning approach shows promise as a supportive, non-invasive diagnostic tool, pending further validation in prospective clinical settings.

## Data Availability

The original contributions presented in the study are included in the article/supplementary material. Further inquiries can be directed to the corresponding author.
